# Development of bilateral chylothorax in a younger female secondary to tuberculosis

**DOI:** 10.4103/0970-2113.76303

**Published:** 2011

**Authors:** Surya Kant, Sanjay Kumar Verma, Sunish C. Anand, Rajendra Prasad, Rajendra Kumar Verma

**Affiliations:** *Department of Pulmonary Medicine, CSM Medical University (Formerly King George’s Medical University), Lucknow, India*; 1 *GSVM Medical College, Kanpur, Uttar Pradesh, India*; 2 *Department of Internal Medicine, Regency Hospital, Kanpur, Uttar Pradesh, India*

**Keywords:** Chylothorax, tuberculosis, analysis

## Abstract

Chylothorax is a rare clinical entity characterized by a milky white aspirate with increased triglyceride levels. The commonest etiology is malignancy and trauma, and bilateral chylothorax, secondary to tuberculosis, is an extremely rare cause, as observed in the present case.

## INTRODUCTION

Chylothorax is a relatively rare cause of a pleural effusion and it occurs when chyle, characterized by high-triglyceride and low-cholesterol concentrations, is found in the pleural space and is usually associated with neoplasms or trauma to the thoracic duct.[1,2] The development of bilateral chylothorax secondary to tuberculosis is a very uncommon clinical entity.[3] Hereby we describe a case of bilateral chylothorax secondary to tuberculosis with cervical lymphadenopathy, in a 15-year-old female.

## CASE REPORT

A 15-year-old female was admitted to our department with the complaints of breathlessness, and off and on fever for 1 month. She came from a nonendemic zone of filariasis in Uttar Pradesh. The resting pulse rate was 92/min and blood pressure was 112/74 mmHg. There was no history of contact with a tuberculosis patient. Her physical examination revealed hard, mobile lymph nodes of size 2 × 2 cm approximately, present bilaterally in the cervical region, and pallor. On chest examination, there was stony dull note localized to the bilateral infrascapular, lower axilla. Rest of the systems were within normal limits.

**Figure 1:**
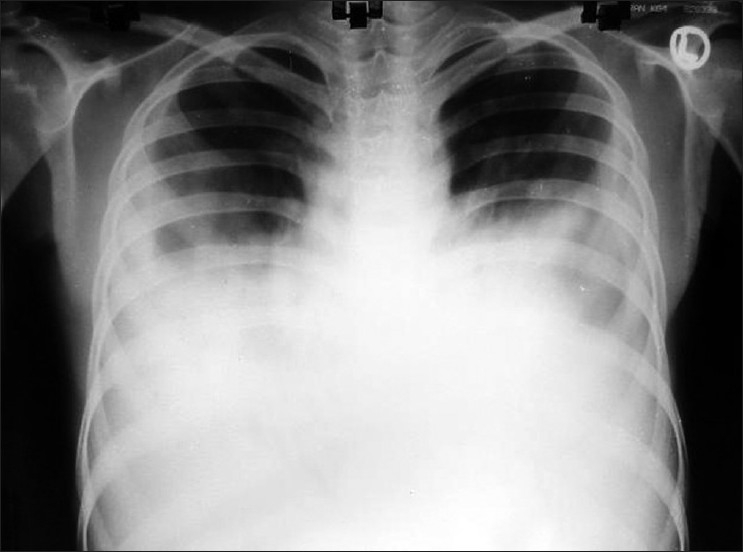
Chest X-ray revealed bilateral pleural effusion

Her blood examination revealed anemia, and hypoalbuminemia but normal total and differential leukocyte counts. Her chest x-ray revealed bilateral pleural effusion [Figure 1]. The pleural fluid was aspirated (about 2.0 l from left and 1.2 l from right) on both sides that revealed a milky white fluid. Then we thought about the chylothorax. Clearing of the pleural fluid by adding ethyl-ether into it leads to the exclusion of pseudochylothorax. The pleural fluid of both sides was sent for examination that revealed, for the right side, protein 4.2 g%, sugar 44 mg%, and total leukocyte count 3400 cells/mm^3^; differential leukocyte count was neutrophils 20, lymphocytes 80, pleural fluid triglyceride 535 mg%, and pleural fluid cholesterol 24.8 mg%; pleural fluid of the left side revealed protein 4.0 g%, sugar 40 mg%, and total leukocyte count 3280 cells/mm^3^; differential leukocyte count was neutrophils 34, lymphocytes 66, pleural fluid triglyceride 188.0 mg%, and pleural fluid cholesterol 24.0 mg% [Table 1]. Serum triglyceride and serum cholesterol was 72.7 mg% and 71.9 mg%, respectively. The Ziehl-Neelsen stain of the pleural fluid was negative but *Mycobacterium tuberculosis* was isolated on the Lowenstein–Jensen culture. The pleural fluid culture for pyogenic organisms was sterile in nature on both sides. PPD showed no indurations. Her biopsy of the cervical lymph node revealed caseating granuloma, and the Bactec culture for *M. tuberculosis* was also positive in the cervical lymph node biopsy specimen. Her abdomen ultrasound also revealed multiple retroperitoneal and para-aortic lymphadenopathy. Thus a diagnosis of bilateral chylothorax secondary to tuberculosis with cervical lymphadenopathy was established.

**Table 1:** Characteristics of the pleural fluid on both sides of the cavity

Pleural fluid	Right pleural cavity	Left pleural cavity
Protein	4.2 g%	4.0 g%
Sugar	44 mg%	40 mg%
TLC	3400 cells/mm^3^	3280 cells/mm^3^
DLC	P20, L80	P34, L66
Pleural fluid triglyceride	535 mg%	188 mg%
Pleural fluid cholesterol	24.8 mg%	24 mg%
Pleural fluid culture	Sterile	Sterile

Serum triglyceride 72.7 mg%; serum cholesterol 71.9 mg%; Ziehl–Neelsen stain of the pleural fluid was negative but *Mycobacterium tuberculosis* was isolated on the Lowenstein–Jensen culture. Biopsy of the cervical lymph node revealed caseating granuloma, and the Bactec culture for *M. tuberculosis* was also positive

To confirm the exact site of the thoracic duct tear, lymphangiography was planned but her parents refused for further investigations.

**Figure 2:**
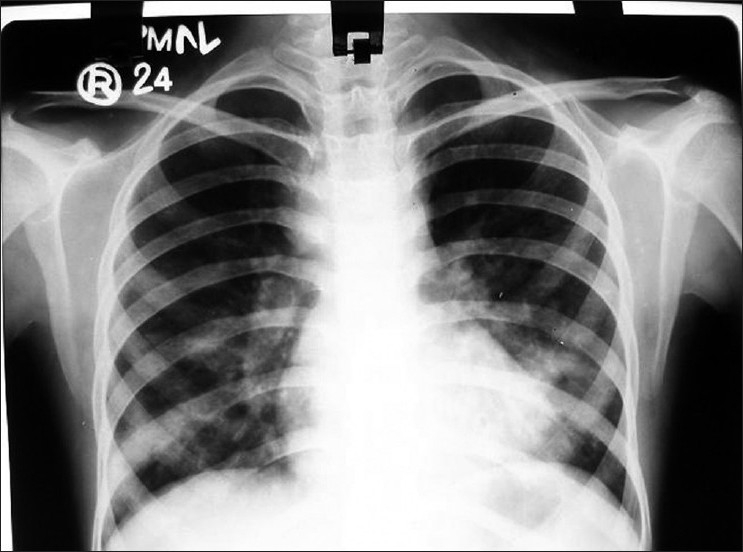
Revealed radiological clearing of the bilateral chylothorax after 2 months of antitubercular treatment

She was put on standard 6-month antitubercular treatment: a combination of isoniazid, rifampicin, pyrazinamide, and ethambutol was started for 2 months followed by isoniazid and rifampicin for a further 4 months. Following this, she showed clinical as well as radiological improvement and chylothorax resolved after 2 months of treatment [Figure 2], and on regular follow-up she had no further symptoms.

## DISCUSSION

Chylothorax was recognized in the seventeenth century but it is still a rare enough entity to be viewed by most physicians as a clinical curiosity. In a historical review by Johsman,[4] Bartolet is credited with the initial description of chylothorax in 1633, and Quincke reported the first case in 1875. Sassion *et al*.[5] divided the causes of chylothorax into four major categories: trauma, tumor, idiopathic, and miscellaneous.

Trauma is the leading cause of chylothorax. This trauma is usually a cardiovascular, pulmonary, or esophageal surgical procedure.[6]

Another leading cause of chylothorax is malignancy. The most common malignancy to cause chylothorax is a lymphoma,[7,8] followed by bronchogenic carcinoma,[9–15] and rarely leukemia. Very few cases of chylothorax secondary to acute lymphoblastic leukemia (ALL) are reported.[16–18]

The third category of chylothorax is idiopathic, including most cases of congenital chylothorax. Most cases of idiopathic chylothorax in adults are probably due to minor trauma,[19] such as coughing or hiccupping after the ingestion of fatty meals.

The fourth category of chylothorax is the miscellaneous category and causes are thrombosis of superior vena cava or subclavian vein,[20] cirrhosis,[21] lymphangioleiomyomatosis,[22] Gorham’s syndrome,[23] Kaposi sarcoma,[24,25] Castleman disease,[26] filirasis and familian lymphedema, sarcoidosis, radiation-induced mediastinal fibrosis, and hypothyroidism.[27]

Tuberculosis is described as a possible cause of chylous effusion,[28] but only a single case, described by Brandt in 1917,[29] appears to have been recorded. Cakir *et al*. reported the concurrence of chlothorax and endobronchial tuberculosis in a 4-month-old boy.[30]

Grobbelaar *et al*. reported one case of bilateral and other case of unilateral chylous effusions associated with extensive mediastinal and hilar lymphadenopathy secondary to pulmonary tuberculosis, in children.[31]

Deniel *et al*. reported spontaneous bilateral chylothorax secondary to disseminated tuberculosis complicated by massive pulmonary embolism.[32]

Tan *et al*. reported a patient with persistent chylothorax and generalized lymphadenopathy who was subsequently diagnosed to have concurrent tuberculosis and malignant lymphoma.[33]

Very few cases of bilateral chylothorax have being reported in the literature.[34] Chylothorax has no predilection for age and sex. Symptoms of chylothorax mostly depend upon the amount of fluid in the pleural cavity.

The exact pathogenesis for the development of chylothorax secondary to tuberculosis remains controversial. Fraser *et al*.[35] and Yunis *et al*.[36] eported that the enlarged lumber and iliac group of lymph nodes produced obstruction of the cisterna chyli and thoracic duct, as a result of which there was dilatation of the lumbar channels; this was followed by the opening up of collateral anastomoses, many lymphaticovenous anastomoses existing between the thoracic duct system and the azygos, intercostal, and lumbar veins. The increased pressure in the system resulted in the transudation of chyle into the pleural space. Grobbelaar *et al*. reported that the possible explanation for the development of a chylothorax in our patients is the obstruction of the thoracic duct by tuberculous lymphadenopathy with subsequent increase in pressure in the surrounding lymphatic system and leaking of chyle into the pleural space.[31]

Best way to establish the diagnosis of chylothorax is to determine the concentration of triglycerides in the pleural fluid. The triglyceride concentration greater than 110 mg/dl (in our case it was 289.3 mg/dl), a ratio of pleural fluid to serum triglycerides of greater than 1.0 (in our case it was 3.77), and a ratio of pleural fluid to serum cholesterol of less than 1.0 (in our case it was 0.344) usually confirm chylothorax. Chylothorax will be excluded if the pleural fluid triglyceride concentration is less than 50 mg/dl. However, in the case of levels from 50 to 110 mg/dl, a lipoprotein analysis of the pleural fluid should be performed, and the demonstration of chylomicrons in the fluid confirms the diagnosis of chylothorax [Table 2].[37]

**Table 2:** Differential points of chylothorax, pseudochylothorax, and empyema, and parental nutrition entering the pleural space via the subclavian line

Parameter	Chylothorax	Pseudochylothorax	Empyema thoracis	Parental nutrition entering the pleural space via the subclavian line
Definition	Presence of chyle in the pleural cavity; chyle contain chylomicrons, triglycerides, and lymphocytes*	Caused by high-lipid levels (cholesterol/lecithin–globulin complexes) in the pleural fluid	Pleural effusion due to bacterial pneumonia	Presence of high triglyceride levels in the pleural cavity
Color	Milky white	Milky white	Milky white	Milky white
Odor	Odorless	Odorless	Foul smelling	Odorless
Clinical onset	Acute	Chronic	Acute	Acute
Most common cause			Bacterial pneumonia	
On centrifugation	Remain opalescent	Remain opalescent	Supernatant part clear	Remain opalescent
Diagnostic criteria				
PF TG PF Cho/serum Cho ratio Pleural fluid culture PCR of the pleural fluid Addition of 1–2 ml of ethyl ether to the pleural fluid				>110 mg/dl
	>110 mg/dl <1.0 Usually negative Usually negative Remain opalescent Ingestion of a fatty meal with lipophilic dye (drug and cosmetic green no. 6, a coal tar dye), followed by thoracocentesis 30–60 min later and if color changed to green fluid, then it also confirms chylothorax	>1.0 Usually negative	Usually negative, even in the case of frank pus in cavity Most specific to detect DNA of various bacterial various populations -	
		Usually negative		
		Clears as cholesterol dissolved		Remain opalescent The presence of chylomicrons in the lipoprotein electrophoresis profile could be traced back to the lipofundin component of parental nutrition

PF TG = pleural fluid triglyceride; PF Cho/serum Cho ratio = pleural fluid cholesterol/serum cholesterol ratio.

* In the case of levels 50–110 mg/dl, a demonstration of chylomicrons in lipoprotein analysis confirms chylothorax. Levels below 50 mg/dl virtually exclude chylothorax

Primary treatment in the case of chylothorax should be directed toward the correction of malnutrition and compromised immunologic status which is due to repeated pleural fluid aspirations of chyle with its high levels of protein, fat, electrolytes, and lymphocytes.[38] The defect in the thoracic duct often closes spontaneously in the case of traumatic injury. In the case of severe dyspnea, the placement of the pleuroperitoneal shunt or chest tube drainage is mandatory.[39] If the chylothorax persists for more than 4 weeks, consideration should be given to surgical exploration with ligation of the thoracic duct.[40]

In our case, diagnosis of chylothorax was established on typical pleural fluid color, high pleural fluid triglyceride level, high ratio of pleural fluid to serum triglyceride, and low ratio of pleural fluid to serum cholesterol. She responded well to antituberculous treatment.

## CONCLUSION

Thus, we should remember that while treating any patient with chylothorax, the probable diagnosis of tuberculosis should be kept in mind.
